# Changes in Pediatric End-of-Life Process After the Enforcement of the Act on Life-Sustaining Treatment Decisions—The Experience of a Single Children’s Hospital

**DOI:** 10.3390/healthcare12212156

**Published:** 2024-10-29

**Authors:** Da-Eun Roh, Jung-Eun Kwon, Young-Tae Lim, Yeo-Hyang Kim

**Affiliations:** 1 Department of Pediatrics, Busan Paik Hospital, Inje University College of Medicine, Busan 47392, Republic of Korea; ponyks1004@naver.com; 2 Department of Pediatrics, School of Medicine, Kyungpook National University, Daegu 41944, Republic of Korea; lovecello623@gmail.com (J.-E.K.); ytlim@knuh.kr (Y.-T.L.); 3 Pediatric Palliative Care Center, Kyungpook National University Children’s Hospital, Daegu 41404, Republic of Korea

**Keywords:** terminal care, palliative care, advance directives, withholding treatment, children

## Abstract

**Background:** The Act on Life-Sustaining Treatment (LST) for patients at the end of life (the Korean LST Decision Act), implemented in the Republic of Korea in February 2018, has led to changes in the end-of-life decision-making (EOLDM) process in children. This study aimed to investigate changes in pediatric EOLDM process and LST practices since the Korean LST Decision Act. **Methods:** This retrospective cohort study included 107 patients who died at Kyungpook National University Children’s Hospital from January 2015 to December 2020. Patients were divided into two groups: pre-law (January 2015–January 2018, *n* = 55) and post-law (February 2018–December 2020, *n* = 52). We analyzed medical records for EOLDM process, patient characteristics, intensive care unit (ICU) admission, documentation types, and LST withholding or withdrawal decisions. **Results:** After the Korean LST Decision Act, the median total hospitalization duration decreased significantly (14 days [IQR, 3–80] vs. 6 days [IQR, 2–18], *p* = 0.020), as did the median ICU length of stay (3 days [IQR 1–33] vs. 2.5 days [IQR 1–10.3], *p* = 0.002). The time from admission to end-of-life decision documentation was significantly shorter in group 2 (6 days [IQR 1–31] vs. 4 days [IQR 1–9], *p* = 0.027). The use of physician orders for life-sustaining treatment (POLST) documents increased (0% to 33.3%), while do-not-resuscitate (DNR) orders decreased (85.3% to 16.7%). Notably, LST withdrawal decisions increased from 0% to 27.8% (*p* = 0.001) in the post-legislation period. **Conclusions:** The Korean LST Decisions Act has led to significant changes in the EOLDM process for terminally ill children, including earlier decision-making, increased use of POLST documents, more frequent LST withdrawal decisions, and shorter hospital and ICU stays. These findings suggest a shift towards more structured and timely end-of-life care discussions in pediatric settings.

## 1. Introduction

For pediatric patients, the continuation of life-sustaining treatment (LST) poses unique ethical dilemmas and is not always advantageous. Maintaining unnecessary LST for patients without the potential for recovery brings about several drawbacks, including extended hospital stays and elevated medical expenses, which subsequently diminish the quality of end-of-life (EOL) care. Moreover, given the constrained resources within the intensive care unit (ICU), perpetuating non-beneficial treatment for terminally ill patients results in an inefficient allocation of medical resources [1].

In Republic of Korea, discussions on how to determine LST use have been ongoing since the late 1990s, and the “Act on Hospice and Palliative Care and Decisions on Life-Sustaining Treatment (LST) (hereafter, Korean LST Decisions Act) for Patients at the End of Life” was enacted in February 2018. The Korean LST Decisions Act ensures the best interests of patients by allowing patients to make their own decisions about LST during the EOL process, protecting the patient’s right to self-determination and legally protecting the patient’s decisions. Informed consent to discontinue LST can be given in one of three ways: first, the patient expresses consent clearly in an advance directive or LST plan; second, if the patient is unconscious, two members of the patient’s family provide consistent statements regarding the patient’s intentions; and third, through the unanimous agreement among the patient’s family members. In the case of patients younger than 18, the decision to terminate LST is made by the patient’s legal representative (limited to those with parental authority). Following the implementation of this legislation, numerous studies on LST were conducted in Korea, with a primary focus on adult patients. Among these adult studies, it was noted that the self-determination rate regarding LST increased by as much as 20–25% compared to studies performed before the legislation came into force [2,3]. Nevertheless, this rate remains comparatively lower than that of Western countries [4]. Furthermore, reports have indicated that across all age groups of adults, family-based determinants outweigh self-determination [3,5,6]. The average duration of hospital stays, admission rates to the ICU, and instances of cardiopulmonary resuscitation (CPR) reduced after the enactment of the law. Additionally, rates of direct transfers to secondary and nursing hospitals increased among adults at the EOL process [7].

While adult studies indicate a growing trend in self-determination regarding LST, pediatric patients often remain subject to familial and systemic influences that may not prioritize their best interests. The decision-making process for pediatric patients is inherently more complex due to issues of legal capacity, parental rights, and the evolving autonomy of the child. Furthermore, there are several factors that make it difficult for parents to make end-of-life decisions (EOLDs): parental need to maintain parent/child relationships, inadequate emotional support, poor communication with healthcare teams, and lack of information of EOLD [8,9]. These factors necessitate a distinct approach to EOL care practices for children.

In the Republic of Korea, there are still numerous cases where potentially futile LST continues during EOL among children and adolescents. This can be attributed to a lack of awareness among family members, confusion, and concerns among medical staff regarding discontinuing LST, as well as systemic limitations and cultural characteristics. In Korean culture, where familial bonds are deeply ingrained, decisions to limit or withdraw treatment may be perceived as abandoning one’s child, leading to a more conservative approach in pediatric EOL care. This passive approach to EOLD for children contrasts with the active approaches of Western countries [10,11,12].

Recently, there has been an increasing interest in pediatric end-of-life decision-making (EOLDM) and demand for pediatric EOLDM guidance [13]. Some pediatric hospice and palliative care centers in Korea are actively trying to apply the Korean LST Decisions Act for pediatric patients in terminal stages.

Several studies have shown that the Korean LST Decision Act has brought about positive changes in the EOLDM process, including increased patient autonomy, earlier decision-making, reduced use of some futile LSTs, and a trend toward lower medical costs. However, there is still a lack of research on changes in childhood EOLD since the enforcement of the Korean LST Decisions Act. This study aims to address this gap by examining the changes in pediatric EOL care resulting from the enactment of the Korean LST Decisions Act, focusing on the experiences within a single children’s hospital. Specifically, we hypothesize that the implementation of the law led to significant changes in the timing, nature, and documentation of EOLD, and the length of hospital and ICU stays in pediatric patients.

By analyzing these changes, we aim to provide valuable insights into the real-world impact of the Korean LST Decisions Act on pediatric EOL care practices. This information is crucial to help policymakers, healthcare providers, and families navigate the complex challenges of pediatric EOLDM within the context of Korean cultural and legal frameworks.

## 2. Materials and Methods

### 2.1. Study Design and Subjects

This retrospective cohort study included patients under the age of 18 who died at Kyungpook National University Children’s Hospital between 1 January 2015 and 31 December 2020. This period was chosen to provide equal observation times before and after the implementation of the Korean LST Decisions Act. The patients were categorized into two groups. Group 1 (pre-law) consisted of 55 patients admitted and deceased from January 2015 to January 2018, while group 2 (post-law) included 52 patients admitted and deceased from February 2018 to December 2020.

The study included all pediatric patients (age < 18 years) who died in the hospital during the study period. Patients who died in the neonatal intensive care unit were excluded due to the unique nature of EOLD in neonates, which often involve different ethical and clinical considerations.

### 2.2. Data Collection

A comprehensive review of the electronic medical records for each patient’s final admission was performed. Data collected included demographics (sex, age), cause of death, pre-existing comorbidities, length of hospitalization, admission to the ICU, and utilization of medical devices (e.g., endotracheal tubes and central venous catheters).

The EOLDM process data included the following information: where the LST decision was made, the place of death within the hospital, duration from admission to LST decision confirmation, duration from document completion to death, the type of document used (e.g., physician orders for LST, POLST, or do-not-resuscitate order), decision to withhold or withdraw LST, and specific medical interventions that were withdrawn or withheld.

### 2.3. Definition of Terminology

The following definitions used in this study were adopted from the initial version of the Korean LST Decisions Act:

- LST: Medical interventions aimed at prolonging life without therapeutic benefits, including but not limited to CPR, hemodialysis, chemotherapy administration, mechanical ventilation, and other life-supporting devices.
- Withholding LST: The decision not to start or escalate LST in patients where such interventions would not provide meaningful benefits.
- Withdrawal of LST: The active discontinuation of LST already being administered, acknowledging that continued treatment is not aligned with the patient’s best interests [14].
- Physician orders for LST (POLST): In Korea, only individuals aged 19 years and above can create an advance directive for LST. For patients under 18, the decision to terminate LST is made by the patient’s legal representative (limited to those with parental authority). The document requires confirmation from two physicians, including the attending physician and a specialist in the relevant field.
- Documentation for “do-not-resuscitate “(DNR)”: The process of obtaining informed consent for a DNR order was established individually by each medical institution. For patients aged under 18, the guardian is responsible for making and documenting the decision regarding resuscitation in the event of a cardiac arrest.

### 2.4. Statistical Analysis

Statistical analyses were conducted using SPSS Statistics for Windows, version 26.0 (IBM Co., Armonk, NY, USA). Continuous variables were expressed as median and interquartile range (25th–75th percentile), and nominal variables were expressed as percentages. The Mann–Whitney U test was used to compare continuous variables between the two groups, while the Fisher exact test was used for categorical variables. A *p*-value of < 0.05 was considered statistically significant.

### 2.5. Ethics Statement

This study was reviewed and received ethical approval from the Institutional Review Board of Kyungpook National University Chilgok Hospital (Approval No. KNUCH 2021-05-030). The requirement for informed consent was waived due to the retrospective nature of the study.

## 3. Results

### 3.1. Demographic and Clinical Characteristics

The demographic and clinical data of the two groups are summarized in Table 1. There were no significant differences in the median age and male-to-female ratio between the two groups. In both groups, the most common primary diagnosis was neurologic and neuromuscular disease (group 1: 41.8%; group 2: 28.8%). The leading causes of death were malignancies (18.2%), metabolic diseases/congenital anomalies (9%), and trauma/accidents (9%) in group 1 and cardiovascular diseases (21.2%) and trauma/accidents (19.2%) in group 2.

Group 2 exhibited significantly shorter total hospitalization durations compared to group 1 (6 days [IQR 2–18] vs. 14 days [IQR 3–80], *p* = 0.020). Similarly, the length of stay in the ICU was significantly shorter in group 2 (2.5 days [IQR 1–10.3] vs. 3 days [IQR 1–33], *p* = 0.002).

### 3.2. The Changes of EOLD

**Figure 1:**
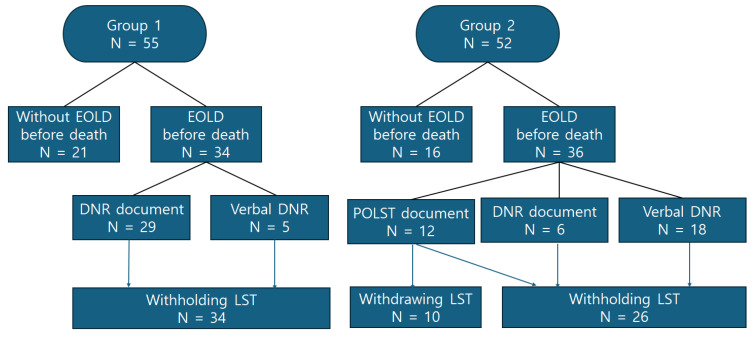
Comparison of end-of-life decision-making processes in two groups. Abbreviations: EOLD, end-of-life decision; DNR, do-not-resuscitate; POLST, physician orders for life-sustaining treatment; LST, life-sustaining treatment.

The details related to EOLD are summarized in Table 2. The proportion of patients who made EOLD before death was not significantly different between the two groups (group 1: 61.8% vs. group 2: 69.2%, *p* = 0.422, Figure 1). In both groups, family members were the decision-makers for EOLD. In group 1, the DNR document was the most frequently used form for EOLD (85.3%). In group 2, 33.3% of the patients used the POLST document. The use of DNR documents decreased to 16.7%, while verbal DNR decisions increased to 50.0% in group 2.

EOLDM processes occurred most frequently in the ICU, followed by the general ward and the emergency department in both groups. The time from admission to the documentation of EOLD significantly decreased (4 days [IQR 1–9] in group 2 compared to 6 days [IQR 1–31] in group 1, *p* = 0.027). The time from EOLD documentation to death was not significantly different between the two groups.

### 3.3. Practical Implementation After EOLD

Before the implementation of the Korean LST Decisions Act, all EOLDs made were about withholding LST. However, after the implementation of the act, 83% of patients who completed POLST documents decided to withdraw their ongoing LSTs (Figure 1).

Among the LSTs that were either withheld or withdrawn, CPR was the most common in both groups, followed by the application of mechanical ventilation and the administration of vasopressors/inotropes (Table 2).

Group 2 with EOLD documentation exhibited significantly shorter total hospitalization durations compared to group 1 with EOLD documentation (6.5 days [IQR 2–21] vs. 15 days [IQR 4–76], *p* = 0.008). However, the length of stay in the ICU did not differ between the two groups.

### 3.4. Comparison between Patients with and Without an EOLD Process in Group 2

As shown in Table 3, there was no significant difference in ICU admission rates according to the presence or absence of EOLD before death in group 2. However, patients with EOLD before death showed a longer hospital stay and significantly longer ICU stay (4 days [IQR 2–12.3] vs. 2 days [IQR 0–3.3], *p* = 0.041) than patients without EOLD before death.

## 4. Discussion

This study observed significant changes in EOLDM processes for pediatric patients following the implementation of the Korean LST Decisions Act. The increased use of POLST documents and the significant rise in LST withdrawal suggest that discussions about EOLD may have become more common in pediatric clinical settings.

Although the Korean LST Decisions Act aimed to focus on patients’ autonomy and dignity in their EOL process, the act does not unconditionally guarantee the autonomy of pediatric patients. This is because while adults aged 18 and above can make EOLDs through advance directives, children and adolescents under 18 cannot be decision-makers even after the Korean LST Decisions Act’s implementation. For this reason, the roles of physicians and families in EOLDM are crucial, and communication between them can have significant impact on EOLDM. Nevertheless, it is a positive step that awareness of the EOLDM process for children has increased after the Korean LST Decisions Act’s implementation. Through active discussions during the EOL phase, physicians can assist families in making decisions regarding withholding or withdrawing LST, potentially helping children avoid futile treatments and receive care with respect and dignity.

Our study revealed differences in the most common diagnosis leading to EOLDM process between adults and children. While cancer is the most common diagnosis among adults [15], neurologic or neuromuscular diseases were the most common among children, followed by hematologic diseases or malignancies in the pre-law period, and cardiovascular diseases and trauma/accidents in the post-law period. Our findings are consistent with results from previous reports on the most common causes of death in children and adolescents with complex chronic conditions [16]. As medical technology advances, children with complex chronic conditions may survive for longer but potentially suffer from chronic pain and frequent hospitalizations. This underscores the importance of accurately determining when a patient is in the terminal stage or at the EOL and initiating timely EOLD discussions.

The transition from written DNR to POLST documents represents a significant improvement in the EOLDM process. Prior to the legislation, DNR forms had limitations due to lack of standardization and legal effect. Before the Korean LST Decisions Act, physicians tended to take a defensive stance out of fear that their current EOLDs could lead to legal issues in the future. The introduction of POLST has provided a more comprehensive and legally recognized approach to the EOLDM process. Interestingly, our findings also showed an increase in verbal DNR decisions, which may be due to patients dying before POLST documentation could be finalized. This reflects the emotional burden parents feel when documenting POLST during the EOLDM process and the need for more time to complete the EOLD.

It is important to note that parents often tend to pursue therapy for their children even when they understand that a cure is unlikely [17,18]. Parents frequently feel guilty when making decisions to withhold or withdraw LST for their children, leading to the continuation of unnecessary LST. In Korea, withholding or minor withdrawal from LST is preferred over complete withdrawal of LST that leads to death within minutes to hours.

Our study demonstrated that after the Korean LST Decisions Act’s implementation, the duration of hospitalization and length of stay in the ICU were significantly shortened. This finding aligns with a population-based study in Korea that reported decreased CPR rates among patients who died in hospitals during the post-legislation period [19]. These results underscore the potential of the Korean LST Decisions Act to minimize unnecessary medical interventions and enhance the effective use of limited medical resources through timely and active discussions on the EOLD [20]. This study is consistent with previous findings in showing a shift toward avoiding futile aggressive treatments during the EOL process in pediatric patients [18].

The ethical and legal distinction between withholding and withdrawing LST is crucial. Prior to the Korean LST Decisions Act, withdrawing LST for children was challenging due to ethical, legal, and cultural factors. The increased willingness to withdraw LST post-legislation represents a significant shift in approach to EOL care for children. However, it is important to note that withdrawal of LST does not equate to cessation of all care; rather, it often signals a transition to palliative care. Post-legislation, patients with an EOLD process stayed in the ICU significantly longer than those who did not. These results are similar to those of other studies analyzing mortality in pediatric ICU [21,22]. This finding suggests that decisions to withdraw or withhold LST tended to occur after a sufficient trial of treatment in the later stages of illness.

Even though the Korean LST Decisions Act has been enacted, it does not mean that EOLDM is appropriately performed in all patients. There is a need for social discussion on EOLDM, education for healthcare providers, education for the public, team-based approaches and support for patients and caregivers during the EOL process, and active communication among healthcare providers, parents, and children.

This study has some limitations. It is a single-center study with a relatively small sample size, which may limit result generalizability. Additionally, the retrospective nature of the study design means we cannot establish causal relationships between the Act’s implementation and the observed changes.

Future research should focus on multi-center studies to confirm these findings across different healthcare settings in the Republic of Korea. Additionally, qualitative research exploring the experiences of healthcare providers and families during the EOLDM process could provide valuable insights for improving EOL care for children.

## 5. Conclusions

In conclusion, this study provides evidence of significant changes in pediatric EOL care following the implementation of the LST Decisions Act in the Republic of Korea. While challenges remain, particularly regarding decision-making involving minors, the Act’s implementation has facilitated more open discussions about EOL care and has potentially improved resource allocation. As we continue to navigate this complex landscape, ongoing evaluation and refinement of policies and practices will be crucial to ensure dignified and appropriate EOL care for all pediatric patients.

## Figures and Tables

**Table 1:** Characteristics of patients.

	Group 1 (N = 55)	Group 2 (N = 52)	*p* Value
Age, year (Median (IQR))	5 (0–12)	3 (0–10)	0.568
Male (%)	25 (45.5%)	33 (63.5%)	0.062
Primary diagnosis (%)			-
Neurologic and neuromuscular disease	23 (41.8%)	15 (28.8%)	
Cardiovascular disease	4 (7.3%)	11 (21.2%)	
Respiratory disease	3 (5.4%)	1 (1.9%)	
Hematologic disease and malignancy	10 (18.2%)	8 (15.4%)	
Gastrointestinal disease	4 (7.3%)	2 (3.8%)	
Metabolic and other congenital anomalies	5 (9.0%)	3 (5.8%)	
Infectious disease	1 (1.8%)	2 (3.8%)	
Renal disease	0 (0%)	1 (1.9%)	
Trauma and accidents	5 (9.0%)	10 (19.2%)	
ICU LOS (days)	3 (1–33)	2.5 (1–10.3)	0.002
Hospital LOS (days)	14 (3–80)	6 (2–18)	0.020
EOLD before death (%)	34 (61.8%)	36 (69.2%)	0.422

Values are presented as median (interquartile range). Abbreviations: ICU, intensive care unit; LOS, length of stay; EOLD, end-of-life decision.

**Table 2:** Comparison of changes in end-of-life decision before and after the enforcement of LST law.

	With EOLD in Group 1 (N = 34)	With EOLD in Group 2 (N = 36)	*p* Value
Place of EOLD			0.176
ICU	23 (67.6%)	30 (83.3%)	
General ward	9 (26.5%)	6 (16.7%)	
Emergency department	2 (5.9%)	0 (0%)	
Time to EOLD after admission (days)	6 (1–31)	4 (1–9)	0.027
Time to death after EOLD (days)	1 (1–31)	2 (1–9)	0.289
Withholding or withdrawing LST			0.001
Withholding	34 (100%)	26 (72.2%)	
Withdrawing	0 (0%)	10 (27.8%)	
Intervention for withholding or withdrawing LST			
Cardiopulmonary resuscitation	34 (100%)	36 (100%)	-
Mechanical ventilation	13 (38.2%)	17 (47.2%)	0.448
Vasopressor or inotropes	12 (35.3%)	10 (27.8%)	0.498
Renal replacement therapy	0 (0%)	3 (8.3%)	0.013
Chemotherapy	0 (0%)	0 (0%)	-
ICU LOS (days)	4 (1–35)	4 (2–12)	0.061
Hospital LOS (days)	15 (4–76)	6.5 (2–21)	0.008

Values are presented as median (interquartile range). Abbreviations: EOLD, end-of-life decision; ICU, intensive care unit; LST, life-sustaining treatment; LOS, length of stay.

**Table 3:** Comparison of end-of-life decision-making status in group 2.

	With EOLD Before Death (N = 36)	Without EOLD Before Death (N = 16)	*p* Value
Age, year (Median (IQR))	3 (0.8–9)	1.5 (0–11.3)	0.965
ICU admission (%)	30 (83.3%)	11 (68.8%)	0.235
ICU LOS (days)	4 (2–12.3)	2 (0–3.3)	0.041
Hospital LOS (days)	6.5 (2–21)	2 (1–16.3)	0.441

Values are presented as median (interquartile range). Abbreviations: EOLD, end-of-life decision; ICU, intensive care unit; LOS, length of stay.

## Data Availability

The de-identified data for this study are available from the corresponding author upon request.
